# Upregulation of bfl-1 is a potential mechanism of chemoresistance in B-cell chronic lymphocytic leukaemia

**DOI:** 10.1038/sj.bjc.6603951

**Published:** 2007-08-28

**Authors:** A Olsson, M Norberg, A ökvist, K Derkow, A Choudhury, G Tobin, F Celsing, A österborg, R Rosenquist, M Jondal, L M Osorio

**Affiliations:** 1 Department of Tumor and Cell Biology, Karolinska Institutet, Stockholm 17177, Sweden; 2 Department of Genetics and Pathology, Rudbeck Laboratory, Uppsala University, Uppsala 75185, Sweden; 3 Department of Clinical Neuroscience, Karolinska University Hospital, Stockholm 17176, Sweden; 4 Department of Oncology–Pathology, Karolinska Institutet, Stockholm 17177, Sweden; 5 Departments of Hematology and Oncology, Karolinska University Hospital, Stockholm 17176, Sweden

**Keywords:** Bfl-1, B-CLL, apoptosis, chemoresistance

## Abstract

B-cell chronic lymphocytic leukaemia (B-CLL) is characterised by the progressive accumulation of monoclonal CD5^+^ B cells. In a previous study, we have analysed the expression profile of apoptosis-regulating genes using a cDNA-based microarray and found overexpression of the antiapoptotic bcl-2 family member, bfl-1, in B-CLL cells with an apoptosis-resistant phenotype. In this study, bfl-1 mRNA levels have been determined by competitive PCR in an extended population of B-CLL patients to characterise its role in disease progression and development of chemoresistance. bfl-1 levels were significantly higher in patients with no response (NR) to last chemotherapy than in patients responding (partial response (PR)) to last chemotherapy (*P*<0.05) and in patients who had not required treatment (*P*<0.05). We found no correlation between bfl-1 mRNA levels and disease progression, IGHV mutational status or other clinical parameters. In addition, bfl-1 mRNA levels were inversely correlated with apoptotic response to *in vitro* fludarabine treatment of B-CLL cells. Specific downregulation of bfl-1 using siRNA induced apoptosis in resistant cells. Our data suggest that bfl-1 contributes to chemoresistance and might be a therapeutic target in B-CLL.

B-cell chronic lymphocytic leukaemia (B-CLL) is characterised by the accumulation of a clonal population of malignant CD5^+^ B cells in the blood, bone marrow, lymph nodes and spleen (Jurlander, 1997). Patients with B-CLL follow a highly variable clinical course. Some patients remain stable for a long time, without the need for therapy, while others progress rapidly to a more advanced disease and die despite aggressive treatment (O'Brien *et al*, 1995). Although this disease is still incurable, responses are observed with alkylating agents or nucleoside analogues. However, despite initial responses to therapy, relapse and eventual chemotherapy resistance usually occur in B-CLL patients.

B-CLL cells have a low proliferative rate and a prolonged life span, suggesting that their accumulation *in vivo* results from defects in the apoptotic process (Hamblin and Oscier, 1997). The underlying defects in apoptosis in B-CLL cells also contribute to chemotherapy resistance. The mechanisms behind the resistance of B-CLL to apoptosis are largely still undefined, but previous studies support a regulatory role of Bcl-2 family of apoptosis-regulatory proteins, such as Bcl-2/Bax and Mcl-1, in the survival of B-CLL cells (Aguilar-Santelises *et al*, 1996; Osorio *et al*, 1997; Kitada *et al*, 1998; Morales *et al*, 2005).

Bfl-1 is an antiapoptotic member of the Bcl-2 family shown to protect from apoptosis induced by a variety of apoptotic stimuli such as death-receptor ligation (Werner *et al*, 2002), p53 overexpression (D'Sa-Eipper *et al*, 1996), DNA-damaging agents (Wang *et al*, 1999) and serum deprivation (Zhang *et al*, 2000). Recently, it was reported that Bfl-1 can also promote apoptosis after TNF receptor activation in pro-B cells, dependent on proteolytic processing of Bfl-1 (Kucharczak *et al*, 2005). This work suggests that Bfl-1 may exert different effects depending on the nature of the death-inducing stimulus. In humans, bfl-1 expression is found in various types of haematopoietic cells in the bone marrow, in germinal centres of peripheral lymphoid organs, haematopoietic cells of fetal liver (Jung-Ha *et al*, 1998), endothelial cells and in smooth muscle cells (Karsan *et al*, 1996). bfl-1 is a direct transcriptional target of NF-*κ*B (Zong *et al*, 1999), and is inducible by inflammatory stimuli in various cell types (Karsan *et al*, 1996; Moreb and Schweder *et al*, 1997). In B lymphocyte-derived cell lines and in primary B cells bfl-1 is induced by CD40 ligation and has been shown to protect from apoptosis triggered by ligation of the B-cell receptor or Fas (Kuss *et al*, 1999; Craxton *et al*, 2000). In B-CLL bfl-1 is inducible by B-cell receptor crosslinking and CD40L (Bernal *et al*, 2001; Kater *et al*, 2004). bfl-1 has also been implicated in CD40L mediated resistance towards fludarabine-induced apoptosis in B-CLL cells (Kater *et al*, 2004). Inhibition of constitutive and inducible NF-*κ*B using NF-*κ*B inhibitors induced apoptosis and enhances fludarabine effects on B-CLL associated with downregulation of bfl-1 and other antiapoptotic genes (Horie *et al*, 2006).

In a previous study, using a cDNA-based microarray approach to characterise expression of apoptosis-regulating genes in B-CLL, we found the antiapoptotic bcl-2 family member bfl-1 as the most discriminating gene between untreated patients whose leukaemic cells were sensitive to *in vitro* fludarabine-induced apoptosis and chemotherapy refractory patients with *in vitro* fludarabine-resistant leukaemic cells (Morales *et al*, 2005). In the present study, bfl-1 mRNA levels have been quantified by competitive PCR in an extended population of B-CLL patients to characterise its role in disease progression and development of chemoresistance. We show a correlation between high bfl-1 expression and chemotherapy refractoriness and resistant to *in vitro* fludarbine-induced apoptosis. In addition, by siRNA-mediated targeting we demonstrate that downmodulation of bfl-1 induces apoptosis in resistant B-CLL cells.

## MATERIALS AND METHODS

### Reagents

Primers for PCR amplification were synthesised by CyberGene AB (Huddinge, Sweden). Trizol Reagent, oligo(dT)_15_ primer and MMLV reverse transcriptase were from Invitrogen (Carlsbad, CA, USA). Fluorescein isothiocyanate (FITC)-F(ab′)2 fragment of rabbit anti-human IgM, phycoerythrin (PE)-conjugated anti-CD25, anti-CD3-PE, anti-CD5-FITC and anti-CD20-PE were from Dakocytomation A/S (Copenhagen, Denmark). Anti-CD38-PE, allophycocyanin (APC) conjugated anti-CD19 and IgG1-FITC/IgG2a-PE simultest were from Becton Dickinson (Mountain View, CA, USA). Fludarabine and chlorambucil were from Sigma Chemicals (St Louis, MO, USA).

### Patients

The study was approved by the Karolinska Institutet Ethics Committee and informed consent was obtained from all patients. No antitumor therapy was allowed for at least 1 month prior to sample acquisition. All patients had a confirmed diagnosis of B-CLL and were staged according to Rai *et al* (1975). Patients were considered to have a progressive disease, according to a modification of the criteria by the National Cancer Institute Committee (Silver *et al*, 1978), if there was progression during the preceding 3 months in disease-related anaemia (and Hb <100 g l^−1^), in thrombocytopaenia (and platelet count <100 × 10^9^ l^−1^) and/or in spleen/liver/lymph node size (evaluated by both clinical examination and computer tomography of the abdomen) and/or in more than a doubling of the blood lymphocyte counts and/or appearance of constitutional symptoms. Response to chemotherapy treatment was classified as no response, partial response or complete response (NR, PR or CR) according to the NCI–WG criteria (Cheson *et al*, 1996); (Table 1).

### Cell separation

Leukaemic B cells were isolated from heparinised blood taken from B-CLL patients. Lymphocytes were obtained after carbonyl iron treatment and Lymphoprep (Nycomed, Oslo, Norway) centrifugation, and T cells were depleted by rosetting with sheep erythrocytes. Isolated cells were kept frozen in aliquots. Isolated non-rosetting, leukaemic B cells contained less than 2.0% CD3^+^ cells as estimated by flow cytometry.

### Cell phenotype

Isolated cells from all B-CLL patients were phenotyped by immunofluorescence and flow cytometry. Cells (1 × 10^6^) were incubated for 30 min at 4°C with anti-CD5-FITC, anti-CD19-APC, anti-CD25-PE, anti-CD20-PE, anti-CD38-PE, anti-CD3-PE or FITC-F(ab′)2 anti-human IgM. FITC/PE-conjugated simultest was used as control. Forward and side-scatter gates were set to exclude dead cells. All samples were analysed in a Becton Dickinson FACScan system equipped with an argon laser, using 10 000 cells for each determination.

### Cell cultures

B-CLL cells were re-suspended in RPMI-1640 medium supplemented with 2 mM glutamine, 100 IU ml^−1^ penicillin, 100 *μ*g ml^−1^ streptomycin and 0.5% bovine serum albumin (BSA fraction-V). Cells (0.5 × 10^6^ ml^−1^) were incubated in 96-well plates in medium alone (spontaneous apoptosis) or in the presence of fludarabine (5 *μ*M) or chlorambucil (40 *μ*M) for 48 h to evaluate apoptotic response. The NIH3T3 mouse fibroblast cell line transfected with human CD40L were cultured in RPMI-1640 medium supplemented with 10% fetal bovine serum (FBS), 2 mM glutamine, 100 IU ml^−1^ penicillin, 100 *μ*g ml^−1^ streptomycin in 24-well plates (2 × 10^5^ cells per well), and incubated overnight to adhere before addition of siRNA-transfected B-CLL cells.

### Apoptosis

Percentage of apoptotic cells was determined by AnnexinV staining. Cells were washed with PBS and incubated 10 min in 100 *μ*l of binding buffer (10 mM HEPES/NaOH, pH 7.4, 140 mM NaCl, 5 mM CaCl_2_) containing AnnexinV-Fluos solution (Roche Molecular Biochemicals, Mannheim, Germany) and 2 *μ*g ml^−1^ propidium iodide (Sigma Chemicals). Cells were analysed with a FACScan (Becton Dickinson). For fludarabine- and chlorambucil-induced apoptosis percentages of specific apoptosis were calculated as (apoptosis in drug culture−spontaneous apoptosis)/(100−spontaneous apoptosis) × 100%.

### Competitive PCR

Total RNA was isolated from purified B-CLL cells using Trizol reagent. Three micrograms of total RNA was denatured and reverse transcribed using oligo-(dT)_15_ primers. Thereafter, 1 *μ*l of cDNA was amplified in a 20 *μ*l PCR mixture. Two microlitres of serial dilutions of competitor fragments, with different lengths but using the same primers as the target DNA, was added to the reaction. *G3PDH* competitor (MIMIC, 630 bp) was from Clontech (Mountain View, CA, USA) and the *bfl-1* competitor (446 bp) was from Gentaur Molecular Products (Brussels, Belgium). Competitor for *bcl-2* (230 bp) was built using composite primers and an exogenous DNA fragment (*Bam*HI–*Eco*RI restriction fragment from *v-erb*). Cycling conditions for bfl-1 and bcl-2 were 30 cycles of 1 min at 94°C, 1 min at 60°C and 2 min at 72°C and for G3PDH 35 cycles of 1 min at 94°C, 1 min at 60°C, and 1 min at 72°C. The samples were then resolved on a 2% agarose gel with 1 *μ*g ml^−1^ of ethidium bromide and photographed. Densitometric analysis was performed using Quantity One (BioRad, Hercules, CA, USA). Ratios of the intensity of the relevant PCR product pairs were plotted against the concentration of the competitor DNA used in a logarithmic plot. The point of intersection in the curve, where the amounts of target and competitor are equal was used to determine the amount of cDNA in the sample. The primers are as follows: sense *bfl-1*, 5′-GGCAGAAGATGACAGACTGTGAA-3′; antisense *bfl-1*, 5′-TGGTCAACAGTATTGCTTCAGGA-3′ (539 bp); sense *bcl-2*, 5′-CGACGACTTCTCCCGCCGCTACCGC-3′; antisense *bcl-2*, 5′-CCGCATGCTGGGGCCGTACAGTTCC-3′ (319 bp); sense *G3PDH*, 5′-TGAAGGTCGGAGTCAACGGATTTGGT-3′; antisense *G3PDH*, 5′-CATGTGGGCCATGAGGTCCACCAC-3′ (983 bp).

### siRNA treatment of B-CLL cells

Transfection of B-CLL cells was performed according to a modified and optimised TransIT-TKO® Transfection Reagent (Mirius Corporation, Madison, WI, USA) protocol for adherent cells. bfl-1-specific siRNA (Dharmacon SMARTpool® siRNA (proprietary target sequence)) and two nonspecific, scrambled siRNA, pooled together as ‘control siRNA’ were ordered from Dharmacon Research Inc. (Lafayett, CO, USA). B-CLL cells (2 × 10^6^) suspended in 200 *μ*l AIM V cell culture medium supplemented with 5% FBS, 100 IU ml^−1^ penicillin and 100 *μ*g ml^−1^ streptomycin were seeded in each well in 48-well plates. For each well 9 *μ*l TransIT-TKO® and 80 pmol siRNA diluted in Opti-MEM® culture medium were added or cells were left untransfected. Six hours after transfection, cells were transferred to hCD40L-expressing mouse fibroblast cultures. At 24 and 48 h after transfection apoptotic response was determined by AnnexinV staining and mRNA expression was analysed by RT–PCR.

### RT–PCR

Total RNA was isolated using RNeasy Mini Kit (Qiagen, Hilden, Germany), and reverse transcribed using N6 and oligo-(dT)_18_ primers. Thereafter, 1 *μ*l of cDNA was amplified in a 20 *μ*l PCR mixture. Cycling conditions were 30 cycles of 30 s at 94°C, 30 s at 60°C and 30 s at 72°C for RPLP0 and 24 cycles of 1 min at 94°C, 1 min at 60°C and 2 min at 72°C for bfl-1. Primers for bfl-1 are as for competitive PCR, and for RPLP0 sense 5′-TTAAACCCCCTCGTGGCAATC-3′; antisense 5′-CCAACTTCCCCCGCATATGA-3′ (293 bp).

### Cytogenetic analysis

To detect the prognostically relevant chromosomal aberrations del(13q), del(11q), trisomy 12 and del(17p), FISH analysis was performed using commercial probes from Abbot Vysis (Stuttgart, Germany) as described previously (Döhner *et al*, 2000). Two-hundred interphase nuclei were analysed for each probe and sample. Samples showing between 10 and 15% cells with a particular aberration were considered as ‘borderline’.

### IGHV gene analysis

Genomic DNA was prepared from leukaemia cells using the QIAamp DNA Mini Kit from Qiagen (Valencia, CA, USA). PCR amplification of the IGH gene rearrangements was performed with either genomic DNA or cDNA using family-specific IGHV (framework region 1) primers together with one consensus IGHJ primer as described previously. The PCR conditions for the IGH analysis was as outlined earlier with minor modifications (Kuppers *et al*, 1993; Willems *et al*, 2000; van Dongen *et al*, 2003). The PCR products were either direct sequenced (35 cases) using the Big Dye Terminator Cycle Sequencing Reaction Kit (Applied Biosystems, Foster City, CA, USA) or subcloning of the PCR product (three cases) was performed using the TOPO TA Cloning Kit For Sequencing (Invitrogen, Paisley, UK). A minimum of six colonies were subsequently sequenced from each cloned sample. The sequences were analysed using an automated DNA sequencer (ABI 3700; Applied Biosystems).

The obtained IG sequences were submitted to different IG sequence databases, International ImMunoGeneTics (IMGT), IgBLAST (National Centre for Biotechnology Information, USA) and JOINSOLVER® (http://joinsolver.niams.nih.gov), and aligned to the most homologous germline IGHV, IGHD and IGHJ genes. Using the classical IGHV homology cutoff value of 98%, cases were divided in unmutated (⩾98% homology to the corresponding germline gene) or mutated (<98% homology) (Table 1). Cases with less than 98% homology only in a non-functional rearrangement were also considered mutated. At least seven nucleotides (allowing one mutation) aligned to the most homologous germline gene were required to identify the IGHD gene segment. The mutated cases were also classified in ‘low mutated’ (between 97.9 and 95% of homology) or ‘high mutated’ (<95% homology). IGHV gene usage was determined in all patients and patients with IGHV3-21 usage were noted separately because of the clinical importance of this group (poor prognosis), regardless their mutational status (Tobin *et al*, 2002).

### Statistical analysis

Estimation of statistical differences between groups was carried out using the Kruskal–Wallis test for comparison between three or more groups, and the Mann–Whitney *U*-test for comparison between two unpaired groups.

## RESULTS

### High expression of bfl-1 correlates with chemotherapy refractoriness of B-CLL

**Figure 1:**
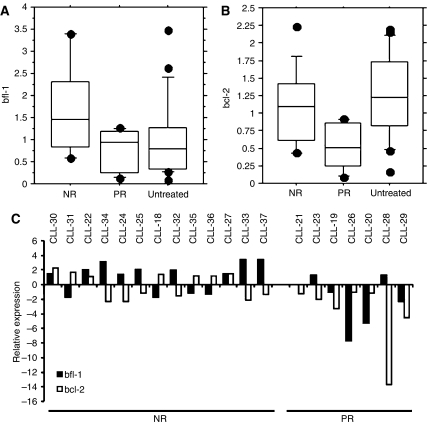
Correlation between bfl-1 and bcl-2 expression and therapy requirement and outcome bfl-1 (**A**) and blc-2 (**B**) mRNA expression was determined using competitive PCR. Results are shown as mRNA expression relative to the median of the sample population. Boxes show intraquartile range. Whiskers correspond to the 10th and 90th percentile, and horizontal line within boxes represents the median value. Data correspond to 17 untreated patients, 13 patients with no response (NR) to treatment and 7 with partial response (PR). bfl-1 comparison NR *vs* PR, *P*<0.05 and NR *vs* untreated, *P*<0.05. bcl-2 comparison NR *vs* PR, *P*<0.05 and PR *vs* untreated, *P*<0.01. (**C**) Comparison of bfl-1 and bcl-2 mRNA expression, as determined by competitive PCR, and therapy outcome. Data are represented as fold increase/decrease mRNA expression relative to the median of the sample population.

bfl-1 mRNA levels were determined by competitive PCR in 37 B-CLL patients, whose clinical characteristics are shown in Table 1, and correlated to *in vivo* response to the last chemotherapy prior to date of sampling (Figure 1A). Twenty of the 37 included patients were treated previously. Last chemotherapy and clinical responses are shown in Table 1. Expression levels of bcl-2 were also determined by competitive PCR and are shown in Figure 1B. bfl-1 expression levels were significantly higher in the NR group (mean=1.77, s.d.=1) compared to the PR group (mean=0.74, s.d.=0.48, *P*<0.05) and compared to the non-treated group (mean=1.0, s.d.=0.89, *P*<0.05). Since most of the treated patients received CLB as the last chemotherapy, the analysis was repeated including only untreated and the 14 CLB-treated patients and equal statistic differences in bfl-1 expression were found as compared to the analysis including all the treated patients (NR *vs* PR *P*<0.05 and NR *vs* untreated *P*<0.05, data not shown). For bcl-2 there was a significant higher expression in the NR group (mean=1.06, s.d.=0.54) compared to the PR group (mean=0.52, s.d.=0.34, *P*<0.05), while the untreated group had levels in the same range as the NR group (mean=1.24, s.d.=0.59) (Figure 1B). However, bcl-2 expression in the untreated group was significantly higher than in the PR group (*P*<0.01). If only CLB-treated patients are included in the analysis, the difference in bcl-2 expression between NR and PR does not reach statistical significance (data not shown). High expression was seen of at least one of the genes, in a majority (10 out of 13) of cases in the NR group, comparing both genes together in the treated patients while the expression of both were in general low in the PR group (Figure 1C).

### High bfl-1 levels correlate with resistance to *in vitro* fludarabine-induced apoptosis

**Figure 2:**
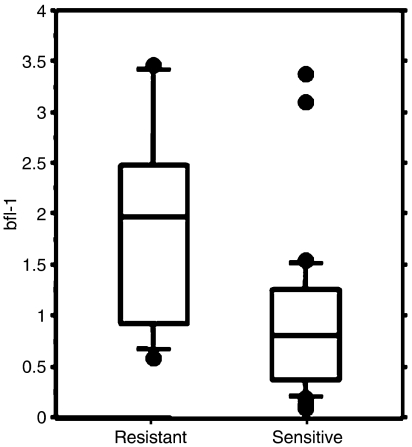
Correlation between bfl-1 expression and *in vitro* fludarabine-induced apoptosis. Isolated B cells (0.5 × 10^6^) from B-CLL patients (all 37 patients included in the study, Table 1) were cultured in 96-well plates in medium alone (non-supplemented with FBS) or with fludarabine (5 *μ*M). Apoptosis was measured after 48 h of culture using AnnexinV staining and fludarabine-specific apoptosis was calculated as described in Materials and Methods section. Cells were considered resistant if specific fludarabine-induced apoptosis was less than 25% (resistant, *n*=11; sensitive, *n*=26). bfl-1 mRNA expression was determined before culture using competitive PCR. Results are shown as mRNA expression relative to the median of the sample population. Boxes show intraquartile range. Whiskers correspond to the 10th and 90th percentile, and horizontal line within boxes represents the median value. Comparison: resistant *vs* sensitive, *P*<0.01.

Leukaemic cells from the patients included were tested for *in vitro* fludarabine- and chlorambucil-induced as well as spontaneous apoptosis. Cells were cultured in the presence or absence of drug (5 *μ*M fludarabine or 40 *μ*M chlorambucil) for 48 h and apoptosis quantified by AnnexinV staining. Specific drug-induced apoptosis was calculated after subtraction of spontaneous apoptosis as described in Materials and Methods section (Table 1). An arbitrary cutoff of 25% specific apoptosis was used to consider patient as resistant or sensitive to *in vitro* drug-induced apoptosis, based on our experience with *in vitro* cultured B-CLL cells. Fludarabine-resistant cells expressed significantly higher levels of bfl-1 mRNA compared to sensitive cells (mean=1.87, s.d.=0.99 and mean=0.91, s.d.=0.81, respectively, *P*<0.01, Figure 2). No correlation between bfl-1 levels and spontaneous apoptosis or chlorambucil-induced apoptosis was found (data not shown). When only the untreated patients (*n*=17, Table 1) were considered, we observed that two samples were resistant to fludarabine and had very high levels of bfl-1, while samples from the rest of the untreated patients were sensitive and expressed low levels of bfl-1 mRNA (*P*<0.05, data not shown). There was no significant correlation between bfl-1 expression and apoptotic response to fludarabine in the treated group (data not shown). No difference in expression of bcl-2 mRNA was detected between cells that were either sensitive or resistant to fludarabine or chlorambucil treatment *in vitro* (data not shown). Cells from NR patients were significantly more resistant to fludarabine than those from PR or untreated patients (*P*<0.01) (data not shown).

### Relationship between bfl-1 expression and disease progression, IGHV mutational status and genetic alterations

Next, we wanted to investigate if bfl-1 was involved in disease progression, and thus bfl-1 mRNA expression was compared between progressive and non-progressive patients. Although there was a tendency to higher bfl-1 expression in progressive patients the difference did not achieve statistical significance (data not shown). In addition, bfl-1 expression did not correlate with lymphocyte count, Rai stage, age, sex or CD38 expression (data not shown).

The IGHV gene mutation status was analysed in 36 B-CLL cases included in this study using IGHV gene family-specific PCR amplification and nucleotide sequencing as described in Materials and Methods section (Table 1). bfl-1 or bcl-2 mRNA expression did not correlate with the mutational status of the patients, independently of cutoff value (98 or 95%) and if the five cases using the VH3-21 gene were considered as a separated group (data not shown).

**Figure 3:**
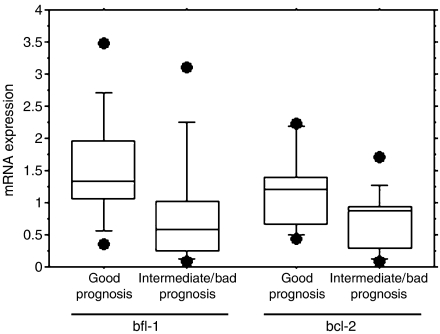
Correlation between bfl-1 and bcl-2 expression and chromosomal aberrations. The prognostically relevant chromosomal aberrations del(13q), trisomy 12, del(11q) and del(17p) were determined by FISH analysis in 28 B-CLL patients included in the study (Table 1). Patients were grouped in good prognosis del(13q)/‘normal’ group (*n*=14) and intermediate/bad prognosis trisomy 12/del(11q)/del(17p) group (*n*=13). bfl-1 and blc-2 mRNA expression was determined using competitive PCR and results are shown as mRNA expression relative to the median of the sample population. Boxes show intraquartile range. Whiskers correspond to the 10th and 90th percentile, and horizontal line within boxes represents the median value. Comparison del(13q)/‘normal’ *vs* trisomy 12/del(11q)/del(17p) for bfl-1, *P*<0.01, and for bcl-2 *P*<0.02.

The prognostically relevant chromosomal aberrations del(11q), trisomy 12, del(13q) and del(17p) were analysed by fluorescence *in situ* hybridisation (FISH) in 28 B-CLL cases. We found three B-CLL patients with no aberration (11) and 11 (39%) with the good prognostic aberration 13q deletion as single aberration, whereas the intermediate prognostic aberration trisomy 12 and the poor prognostic aberrations 11q deletion and 17p deletion were detected in 4 (14), 5 (18) and 4 (14%) patients, respectively. Among the patients with 17p deletion one was considered borderline (10–15% cells with the aberration). In one patient (3%), the FISH data were not conclusive (Table 1). bfl-1 or bcl-2 mRNA was significantly higher expressed in the good prognosis del(13q)/‘normal’ group as compared to the intermediate/bad prognosis trisomy 12/del(11q)/del(17p) group (*P*<0.01 and *P*<0.02, respectively) (Figure 3). bfl-1 or bcl-2 mRNA expression was also compared, within each cytogenetic group (good and intermediate/bad prognosis), between NR and PR patients, and between treated and untreated patients. Although this study included too few patients to make a proper analysis and draw any conclusions, we noted that both bfl-1 and bcl-2 levels were higher in NR patients than in PR patients, independent of the cytogenetic status. However, there were no differences between treated and untreated patients within each cytogenetic group (data not shown).

### siRNA-mediated downregulation of bfl-1 induces apoptosis in resistant B-CLL cells

**Figure 4:**
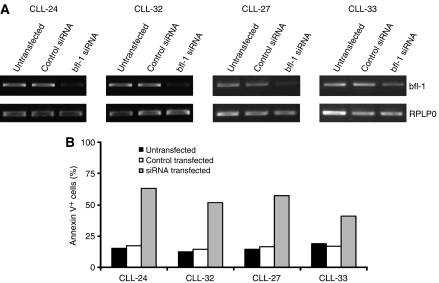
siRNA-mediated targeting of bfl-1 in B-CLL cells. B-CLL cells from four *in vitro* fludarabine-resistant and bfl-1 high-expressing cases (CLL-24, CLL-32, CLL-27 and CLL-33) were transfected with bfl-1-specific siRNA, control siRNA oligo or were not transfected. At 24 h cells were harvested, mRNA was isolated and analysed for bfl-1 mRNA expression by RT–PCR. (**A**) At the same time apoptosis was quantified using AnnexinV staining to quantify apoptosis. (**B**) Data show one representative experiment out of two.

To test directly the contribution of blf-1 to apoptosis resistance in B-CLL we targeted bfl-1 expression by specific siRNA in fludarabine-resistant, bfl-1 high-expressing B-CLL cells (CLL-24, CLL-32, CLL-27 and CLL-33; Figure 4). Initial experiments with fluorescently labelled nonspecific siRNA showed efficient uptake in B-CLL cells under the conditions used (Derkow, unpublished observation). Downmodulation of bfl-1 mRNA was seen 24 h after transfection (Figure 4A) and expression levels did not decrease further at 48 h (not shown). At 24 h apoptosis was also clearly induced in cells transfected with bfl-1-specific siRNA but not in cells transfected with control siRNA or in untransfected cells (Figure 4B) and did only further increase slightly at 48 h (not shown), showing that in these resistant cases with high bfl-1 expression, bfl-1 is indeed important for the survival of the cells.

## DISCUSSION

Even though the majority of symptomatic B-CLL patients respond to initial therapy, almost all progress and develop refractory disease given sufficient time. The underlying defects in apoptosis in B-CLL cells presumably contribute to the chemotherapy resistance in such patients. Here, we have shown high bfl-1 mRNA expression in leukaemic cells from chemotherapy refractory B-CLL patients. Several members of the Bcl-2 family of apoptosis-regulating proteins have previously been implicated in the deregulation of apoptosis and in chemotherapy resistance in B-CLL (Morales *et al*, 2005). Higher levels of Mcl-1 have been reported to be associated with a failure to achieve complete remission following chemotherapy (Kitada *et al*, 1998). Higher levels of Bcl-2 protein or higher Bcl-2/Bax ratios have been associated with *in vitro* resistance to drug-induced apoptosis (McConkey *et al*, 1996; Thomas *et al*, 1996). Increased Bcl-2/Bax ratios have also been correlated to a more aggressive behaviour of B-CLL, including progressive disease, refractoriness to chemotherapy, and shorter survival (Aguilar-Santelises *et al*, 1996, Pepper *et al*, 1998). However, not all studies have identified an association between Bcl-2 or Bax expression and chemoresistance and/or clinical outcome in B-CLL, implying that other factors play a role (Robertson *et al*, 1996; Johnston *et al*, 1997; Kitada *et al*, 1998). Using microarrays, we recently found a pattern of high expression levels of bfl-1, bcl-2 and mcl-1 mRNAs in apoptosis-resistant B-CLL cells (Morales *et al*, 2005). In our current study, we found significantly higher expression of bfl-1 and bcl-2 in the refractory patients. The strongest difference between non-responding and responding patients was seen when both bfl-1 and bcl-2 were considered together, with high expression of at least one of the genes in the refractory group compared to low expression of both in patients that had responded to chemotherapy. This indicates that the expression of both bfl-1 and bcl-2, and possibly additional members of the bcl-2 family, such as mcl-1, may give the best prediction of chemotherapy response.

Our current results do not answer if high mRNA expression levels of bfl-1 is predictive of chemotherapy outcome or if it is a result of resistance development. The fact that most untreated patients had low bfl-1 levels and that the majority of the treated patients had received several rounds of treatment, responding initially, argues that bfl-1 expression could be increased as a result of treatment and might contribute to resistance development. In fact, bfl-1 has previously been implicated in chemotherapy resistance development in model systems. Increased expression of bfl-1 has been found in an *in vivo* established vinflunine-resistant murine leukaemia cell line as well as in *in vitro* established cisplatin-resistant bladder cancer cell lines compared to their respective sensitive parental cell lines (Kruczynski *et al*, 2002; Kim *et al*, 2004). We have found that fludarabine did not induce increased bfl-1 expression in B-CLL cells in a short-term culture (Olsson, unpublished observation). However, most patients had been receiving other chemotherapy regimens and the increased bfl-1 expression could be a long-term effect or due to selective killing of cells expressing lower levels of bfl-1. Prospective studies are warranted to resolve this question.

We did not find any correlation between high expression of bfl-1 and progressive disease or advanced stage of disease. This indicates that at least in part different factors govern disease progression/aggressiveness of disease and chemotherapy refractoriness. Absence of somatic IGHV hypermutations has been associated with the requirement of therapy and poor survival (Damle *et al*, 1999; Hamblin *et al*, 1999). Although no study investigating the impact of IGHV mutation status on the outcome of therapy of B-CLL patients has been reported, an *in vitro* study shows higher sensitivity to induction of apoptosis by several chemotherapeutic agents in unmutated cases (Åleskog *et al*, 2004). This, together with our results of no correlation of bfl-1 or bcl-2 antiapoptotic gene expression with the mutational status, suggest that the difference in prognosis between B-CLL cases with unmutated or mutated IGHV genes is not explained by the cellular apoptosis sensitivity.

It was surprising to find a higher expression of bfl-1 mRNA in patients with del(13q) or ‘normal’ karyotype, as compared to the trisomy 12/del(11q)/del(17p) group. As a single cytogenetic defect, del(13q) is a good prognosis marker, associated with longer overall and treatment-free interval (Döhner *et al*, 2000). However, there is no study on the prognostic significance of del(13q) on the response to therapy. Others have found higher bcl-2 expression in B-CLL cells with del(13q) or normal karyotype as compared to the bad prognosis groups del(17p), trisomy 12 or del(11q), which is in agreement with our results (Jahrsdörfer *et al*, 2005). In addition, the expression of two microRNAs, miR-15a and miR-16-1, is inversely correlated to bcl-2 expression in B-CLL samples and they negatively regulate bcl-2 at a post-transcriptional level (Cimmino *et al*, 2005). These two microRNAs are located in the minimally deleted region at 13q14.3, which could explain the high expression of bcl-2 in the del(13q) group. Further studies are ongoing to explore the relation between chromosomal aberrations and apoptosis-regulating genes in B-CLL, as well as to determine the predictive value of apoptosis-regulating genes for the treatment response in good and bad prognosis cytogenetic groups.

We found a correlation between bfl-1 expression and *in vitro* fludarabine-induced apoptosis, but not with spontaneous or chlorambucil-induced apoptosis. However, bfl-1 expression correlated with *in vivo* response to chlorambucil. Previously, Mcl-1 expression was reported to correlate to *in vitro* chlorambucil-induced apoptosis but not to fludarabine-induced apoptosis (Johnston *et al*, 2004). Although both drugs induce apoptosis through a mitochondria-dependent pathway, initiation of the pathway may depend on different mechanisms, which might be selectively counteracted by bfl-1 or mcl-1. In contrast to what is the case for the proapoptotic bcl-2 family members, not much is known about functional differences between individual antiapoptotic members of the family. Growing evidence indicates, however, that different antiapoptotic bcl-2 family members are counteracted specifically by different proapoptotic BH3-only proteins activated by different signals (Chen *et al*, 2005; Willis *et al*, 2005). Further studies are bound to find other functional niches for other antiapoptotic bcl-2 members, which might explain their selective protection against various apoptotic stimuli activating the mitochondrial pathway. Our contradictory results regarding the correlation of bfl-1 expression level with *in vitro* and *in vivo* response to chlorambucil might also be explained by differences in the mechanisms regulating the apoptotic process *in vitro* and *in vivo*.

By selectively downmodulating bfl-1 using specific siRNA we could induce apoptosis in fludarabine-resistant B-CLL cells, showing that bfl-1 has a protective role against apoptosis in these cells. Together with our recent finding that bfl-1 mRNA expression levels are decreased in apoptosis-sensitive cells during spontaneous apoptosis *in vitro* (Morales *et al*, 2005) and the finding by Kater *et al* (2004) that CD40L, known to promote survival of B-CLL cells *in vivo*, induces the expression of bfl-1 in B-CLL cells, protecting them from spontaneous and fludarabine-induced apoptosis *in vitro*, this finding suggests that bfl-1 may be important for the extended survival of the leukaemic cells *in vivo*.

Targeting bcl-2 family members is recognised as a promising therapeutic strategy in B-CLL. bcl-2 antisense therapeutic strategy has proven feasible without toxicity and is already in clinical trials for B-CLL patients (O'Brien *et al*, 2005). Our results suggest that an approach involving also the targeting of bfl-1 deserves further testing.

## Figures and Tables

**Table 1:** Clinical characteristics, apoptotic response, mutational status and genetic alterations of included patients

**CLL**	**Sex/age**	**Rai stage**	**Progression**	**Mo since diagnosis**	**Previous therapy^a^**	**Blood lymphocyte count (× 10^9^ l^−1^)**	**Fludarabine-specific apoptosis (%)**	**CLB-specific apoptosis (%)**	**Response to therapy**	**FISH code^b^**	**IGHV mutational status^c^/IGHV3-21^d^**
1	M/73	0	—	53	—	40	24.0	59.1	—	del(13q)	Mutated (high)
2	M/66	0	—	68	—	20	90.4	98.4	—	ND	Mutated (high)
3	F/61	0	—	135	—	78	82.0	84.6	—	del(13q)	Mutated (high, NF)
4	M/67	0	—	90	—	22	88.1	94.9	—	Normal	ND
5	M/86	0	—	39	—	55	80.4	26.4	—	del(11q)	Mutated (high)
6	F/76	0	—	47	—	62	75.7	59.4	—	Normal	Mutated (high)
7	F/81	0	—	179	—	104	89.0	89.1	—	trisomy 12	Mutated (low)
8	M/69	0	—	78	—	25	82.6	45.8	—	del(13q)	Mutated (low)
9	M/69	0	—	26	—	74	42.5	67.6	—	ND	Unmutated
10	M/58	0	—	30	—	35	88.4	ND	—	ND	Mutated (high)
11	M/59	0	—	76	—	27	13.4	18.7	—	del(13q)	Mutated (high, NF)
12	M/76	0	—	32	—	147	71.5	78.3	—	del(11q)	Mutated (high)
13	M/71	I	—	10	—	30	79.8	ND	—	ND	Unmutated
14	F/70	I	—		—	112	73.6	72.4	—	del(13q)	Mutated (low)
15	F/73	I	—	77	—	27	85.1	96.3	—	ND	Mutated (high, NF)
16	M/68	I	—	5	—	42	76.9	39.1	—	del(11q)	Unmutated
17	M/66	II	—	72	—	41	71.2	ND	—	ND	Mutated (high)
18	F/79	II	—	68	CLB (9)	48	44.9	79.4	NR	del(13q)	Mutated (high)
19	M/85	III	—	150	CLB (13)	138	68.5	55.9	PR	del(11q)	Mutated (low)
20	F/77	0	+	122	CLB (6)	38	65.3	50.9	PR	trisomy 12	Mutated (low) IGHV3-21
21	F/79	I	+	53	CLB (6)	61	69.0	81.2	PR	del(13q)	Mutated (low)
22	F/69	II	+	58	CLB (1)	54	7.4	ND	NR	del(13q)	Mutated (low)
23	M/86	II	+	29	CLB (5)	117	82.8	55.7	PR	del(13q)	Unmutated IGHV3-21
24	M/65	II	+	29	CLB (1)	329	21.8	−5.5	NR	Normal	Mutated (low)
25	M/70	II	+	96	CLB (72)	66	11.7	13.8	NR	del(17p)-borderline	Unmutated
26	M/61	II	+	61	FLUD (38)	53	40.6	32.4	PR	del(17p)	Mutated (high)
27	F/87	II	+	260	COP (12)	176	18.6	−1.3	NR	Inconclusive	Mutated (high, NF) IGHV3-21
28	F/80	II	+	69	CLB (8)	208	85.8	18.0	PR	trisomy 12	Mutated (high)
29	M/81	II	+	24	CLB (5)	200	66.4	19.7	PR	del(11q) (& trisomy 12)	Mutated (low) IGHV3-21
30	M/64	III	+	23	FLUD (11)	354	32.3	−2.5	NR	del(13q)	Mutated (low, NF)
31	M/67	III	+	142	MIME (7)	304	3.5	7.1	NR	del(17p)	Unmutated
32	F/69	III	+	239	FLUD (11)	198	7.9	5.3	NR	del(13q)	Mutated (high)
33	F/80	III	+	216	CLB (1)	176	16.4	26.3	NR	ND	Unmutated
34	F/52	IV	+	116	CLB (19)	69	92.5	96.9	NR	trisomy 12	Mutated (high)
35	M/77	IV	+	169	CLB (3)	488	16.7	5.2	NR	del(17p)	Unmutated
36	M/55	IV	+	96	MIME (3)	68	12.9	18.9	NR	ND	Unmutated
37	F/64	IV	+	38	CLB (11)	27	65.8	39.9	NR	ND	Unmutated IGHV3-21

CLB=chlorambucil; COP=cyclophosphamide, vincristine, prednisone; CR=complete response; FLUD=fludarabine; MIME=mitoguanozone, ifosfamide, methotrexate, etoposide; ND=not determined; NF=non-functional; NR=no response; PR=partial response.

a Last therapy before sample and months since last treatment in parentheses. .

b Coding after FISH analysis based on clinical relevance. Del(13q) corresponds to single aberration.

c Unmutated ⩾98% homology, mutated < 98% homology, low: 2–5% mutations, high: >5% mutations.

d IGHV gene usage was analysed in all patients, only IGHV3-21 is noted separately because of the clinical importance, regardless mutational status.
